# Ginsenoside Rg4 Enhances the Inductive Effects of Human Dermal Papilla Spheres on Hair Growth Via the AKT/GSK-3β/β-Catenin Signaling Pathway

**DOI:** 10.4014/jmb.2101.01032

**Published:** 2021-05-31

**Authors:** Yun Hee Lee, Hui-Ji Choi, Ji Yea Kim, Ji-Eun Kim, Jee-Hyun Lee, So-Hyun Cho, Mi-Young Yun, Sungkwan An, Gyu Yong Song, Seunghee Bae

**Affiliations:** 1 Research Institute for Molecular-Targeted Drugs, Department of Cosmetics Engineering, Konkuk University, Seoul 05029, Republic of Korea; 2 College of Pharmacy, Chungnam National University, Daejon 34134, Republic of Korea; 3 Department of Beauty Science, Kwangju Women’s University, Gwangju 62396, Republic of Korea

**Keywords:** Ginsenoside Rg4, hair growth property, dermal papilla, AKT, β-catenin

## Abstract

Ginsenoside Rg4 is a rare ginsenoside that is naturally found in ginseng, and exhibits a wide range of biological activities including antioxidant and anti-inflammatory properties in several cell types. The purpose of this study was to use an in vivo model of hair follicle (HF)-mimic based on a human dermal papilla (DP) spheroid system prepared by three-dimensional (3D) culture and to investigate the effect of Rg4 on the hair-inductive properties of DP cells. Treatment of the DP spheroids with Rg4 (20 to 50 μg/ml) significantly increased the viability and size of the DP spheres in a dose-dependent manner. Rg4 also increased the mRNA and protein expression of DP signature genes that are related to hair growth including *ALP*, *BMP2*, and *VCAN* in the DP spheres. Analysis of the signaling molecules and luciferase reporter assays further revealed that Rg4 induces the activation of phosphoinositide 3-kinase (PI3K)/AKT and the inhibitory phosphorylation of GSK3β, which activates the WNT/β-catenin signaling pathway. These results correlated with not only the increased nuclear translocation of β-catenin following the treatment of the DP spheres with Rg4 but also the significant elevation of mRNA expression of the downstream target genes of the WNT/β-catenin pathway including *WNT5A*, *β-catenin*, and *LEF1*. In conclusion, these results demonstrated that ginsenoside Rg4 promotes the hair-inductive properties of DP cells by activating the AKT/GSK3β/β-catenin signaling pathway in DP spheres, suggesting that Rg4 could be a potential natural therapy for hair growth.

## Introduction

In mammals, hair has various important roles, including the regulation of body temperature and providing physical protection from the external environment [1, 2]. During postnatal development, HFs undergo a cyclical process of hair growth involving three distinct phases, namely, the anagen (active growing stage), catagen (regression stage), and telogen (resting stage) phases [3]. Each phase of the hair cycle results from the compositive interactions between the epithelial cells and specialized mesenchymal cells called dermal papilla (DP), that are located at the base of the HFs [4, 5]. Hair loss or alopecia, is a complex biological process that affects millions of people worldwide, by affecting the social value and psychological health of the individual [6]. Recent studies have demonstrated that multiple causative factors, including hormonal imbalance, psycho-emotional stress, oxidative damage, and environmental assaults, induce various types of hair loss by miniaturization of the hair follicle (HF) via aberrant regulation of the hair cycle [7]. The US Food and Drug Administration (FDA) approved only two medications, finasteride and minoxidil, for the treatment of alopecia [8]. However, these medicines have limited efficacy owing to the long duration of therapy, relapse, and adverse effects [9]. Therefore, the development of an alternative natural therapy with fewer side effects that can prevent hair loss or promote hair growth has been attempted long since.

DP cells are crucial for hair induction, growth, and hair cycling as they generate instructive signals that induce the proliferation and differentiation of epithelial cells [2, 10]. Of the numerous signals, the simultaneous upregulation of the canonical Wnt/b-catenin signaling pathway is essential for maintaining the hair-inductive activity of DP cells [11]. Several studies have also reported that the proliferation and migration of DP cells which affect the regeneration and growth of HFs can be upregulated by the activation of ERK and AKT, and stimulation of the phosphoinositide 3-kinase (PI3K)/AKT pathway [12, 13]. Therefore, numerous studies have frequently utilized DP cells for investigating the mechanism underlying the hair cycle and for the development of drugs for inducing hair growth [14]. Additionally, recent studies have developed human DP cell-based in vitro models using three-dimensional (3D) spheroid culture systems for studying hair growth and hair loss [15]. The studies demonstrated that the DP spheroids in the 3D culture system can rescue the hair-growth inductive properties of DP cells in vivo and restore the expression of signature markers that induce hair regeneration and growth, including alkaline phosphatase (ALP), versican (VCAN), and bone morphogenetic protein (BMP), which are either slightly expressed or absent in two-dimensional (2D) monolayer cultures [16].

Ginseng has a wide range of pharmacological properties including anti-diabetic, anti-cancer, and anti-inflammatory activities [17-19]. Ginsenosides are the major active constituents of ginseng and can be classified into protopanaxadiol (PPD) and protopanaxatriol (PPT) type ginsenosides according to the number of hydroxyl groups that can undergo glycosylation and the steroidal structure [18]. The PPD-type ginsenosides include Rb1, Rb2, Rb3, Rc, Rd, F2, and Rg3, while the PPT-type ginsenosides include Re, Rg1, Rg2, Rg4, Rh1, and Rh4 [20]. Furthermore, single ginsenosides possess diverse beneficial biological activities that find use in cosmetology including increased collagen synthesis [21], antioxidant effects [22], anti-wrinkle effects [23], and the induction of hair growth [24]. As the major low-polarity PPT-type ginsenoside, Rg4 is one of the most effective steroidal saponins among these ginsenosides. Recent studies have reported that the rare ginsenoside Rg4 possesses a wide range of therapeutic and pharmacological properties, including the prevention of disorders related to cartilage degradation [25], anti-cancer activities [26], and anti-inflammatory properties [27]. However, the effects of Rg4 on human hair cells and har growth are yet to be elucidated.

In this study, we evaluated the in vitro efficacy of ginsenoside Rg4 on the hair-inductive properties of DP spheres prepared using a 3D-spheroid culture system. The results indicated that Rg4 could be a naturally occurring therapeutic candidate for hair loss.

## Materials and Methods

### Cell Culture and Reagents

Human follicular DP cells were purchased from PromoCell (Germany) and cultured in Follicle DP Cell Growth Medium kit (PromoCell) at 37°C in a humidified atmosphere of 5% CO_2_ in an incubator. The medium was changed every 3 days, and the cells were harvested with Accutase cell detachment solution purchased from Merck Millipore (USA). Additionally, 293T cells were purchased from American Type Culture Collection (USA). Dulbecco’s modified Eagle’s medium (DMEM; Thermo Fisher Scientific, USA) was supplemented with 5% (v/v) fetal bovine serum (FBS) purchased from Sigma-Aldrich, USA. LY294002, a selective phosphatidylinositol 3-kinase (PI3K) inhibitor, and ginsenoside Rg4, derived from black ginseng, were purchased from Merck (Germany) and AREZ Co. Ltd., (Korea), respectively. The T cell-specific transcription factor and lymphoid enhancer-binding factor (TCF/LEF) luciferase reporter plasmids were purchased from Promega (USA). Minoxidil was purchased from Sigma-Aldrich.

### Culture of DP Cells Using a 3D Spheroid System

The 3D cultures of the DP cells were prepared as previously described [28]. Briefly, the DP cells were seeded in clear 96-well, round-bottomed, ultra-low attachment cell culture microplates (Corning Incorporated, USA) at a density of 8 × 10^3^ cells per well. After the formation of unified spheres, the spheres were treated with Rg4 for 48 h, and the diameter of the spheroids was quantified by comparing with that of the control.

### Cell Viability Assay

The cytotoxicity of Rg4 in DP cells was analyzed using the water-soluble tetrazolium salt (WST-1) assay (EZ-Cytox Cell Viability Assay Kit; Korea). Briefly, the cells (8 × 10^3^ cells/well) were seeded on a 96-well plate and a clear 96-well, round-bottomed, ultra-low attachment microplate and cultured in complete media for 24 h. The cells cultured in the 2D monolayer and the DP spheroids prepared by 3D culture were treated with the indicated doses of Rg4. After an additional 48 h of incubation, WST-1 solution was added to each well and incubated for 0.5 h to allow the formation of formazan. Cell viability was determined by measuring the absorbance at 450 nm using an iMark microplate reader (Bio-Rad Laboratories, USA).

### Total RNA Isolation and Real-Time qPCR

The total RNA was isolated using Ribo EX reagent (GeneAll, Korea). The complementary DNA (cDNA) was synthesized using 1 μg of total RNA, oligo dT, 1.5 mM dNTP, 0.1M DTT, 5 × First Stand buffer, and M-MLV reverse transcriptase (Invitrogen, USA). Real-time qPCR was performed using the StepOnePlus Real-Time PCR system (Thermo Fisher Scientific). The time of amplification by PCR was measured using SYBR Green PCR Master Mix (Thermo Fisher Scientific). The relative gene expression was quantified using the 2^−ΔΔCt^ method and normalized to that of the housekeeping gene, *glyceraldehyde-3-phosphate dehydrogenase* (*GAPDH*). Each of the samples were analyzed by three independent experiments. The sequences of the primers are provided in Table 1.

### Western Blot Analysis

The total cell lysate was prepared with sodium dodecyl sulfate (SDS) lysis buffer (1% [w/v] SDS, Tris-HCl [pH 6.8] buffer, 4% β-mercaptoethanol), containing a protease inhibitor cocktail (Promega) and a phosphatase inhibitor cocktail (Sigma, Switzerland). Then, the same amount of protein lysates were fractionated by 10% SDS-polyacrylamide gel electrophoresis (SDS-PAGE). The protein bands were transferred onto a nitrocellulose membrane over a transfer time of 1 h, and the membrane was blocked with 5% non-fat milk for 1 h. The membrane was subsequently washed thrice and incubated overnight with the primary antibodies at 4°C. The following primary antibodies were used for immunoblotting: β-actin (1:12,000 dilution) purchased from Sigma-Aldrich; BMP2 (ab14933, 1/1,000 dilution), anti-versican (ab19345, 1/500 dilution), ALP (ab108337, 1/2,000 dilution), PI3K p85 (ab86714, 1/1,000 dilution), and β-tubulin (ab6046, 1/1,000 dilution) purchased from Abcam (UK); p38 (sc-728, 1/500 dilution) and lamin A/C (sc-7293, 1/1,000 dilution) purchased from Santa Cruz (Bolivia), phospo-p38 (#9211, 1/1,000 dilution), phospho-ERK (#9101, 1/1,000 dilution), ERK (#9102, 1/1,000 dilution), phospho-JNK (#9251, 1/1,000 dilution), JNK (#9252, 1/1,000 dilution), phospho-PI3K (#4228, 1/200 dilution), phospho-AKT S473 (#9271, 1/300 dilution), AKT (#9272, 1/1,000 dilution), phospho-GSK3β (#9323, 1/1,000 dilution), GSK3β (#9315, 1/1,000 dilution), and β-catenin (#9562, 1:1,000 dilution) purchased from Cell Signaling Technology (UK). Following incubation with the primary antibodies, the membranes were incubated with the secondary antibodies against the primary antibodies for 1 h. Nuclear/cytoplasmic fractionation assays were performed using NE-PER Nuclear and Cytoplasmic Extraction Reagents (Thermo Fisher Scientific). The protein bands were detected by visualization using the ChemiDoc Touch Imaging System (Bio-Rad) with ECL reagent (Bio-Rad).

### Luciferase Reporter Assay

The cells were co-transfected with TCF/LEF luciferase reporter plasmids and pSV-β-galactosidase (pSV-β-gal) plasmids using Lipofectamine 3000 (Invitrogen) as the transfection reagent. After 48 h of incubation, the transfected DP cells were lysed using passive lysis buffer (Promega). For detecting the luciferase activity, the lysates were incubated with D-luciferin (Sigma-Aldrich), and the luciferase activity was measured using a Glomax 96 Microplate Luminometer (Turner BioSystems, USA). The activity of β-gal was analyzed using a Luminescent β-galactosidase Detection Kit II (Clontech Laboratories Inc., USA). The relative luciferase activity was determined by normalizing the luciferase activity to that of β-gal. The results are presented as the mean ± standard deviation (SD) of data obtained from three independent experiments.

### Statistical Analyses

All the data are presented as the mean ± SD of three independent experiments. The normally distributed data were evaluated using a two-tailed Student’s t-test, as depicted in the figures. The differences were considered to be statistically significant when *p < 0.05.

## Results

### Ginsenoside Rg4 Increases Viability and Formation of DP Spheres

**Fig. 1:**
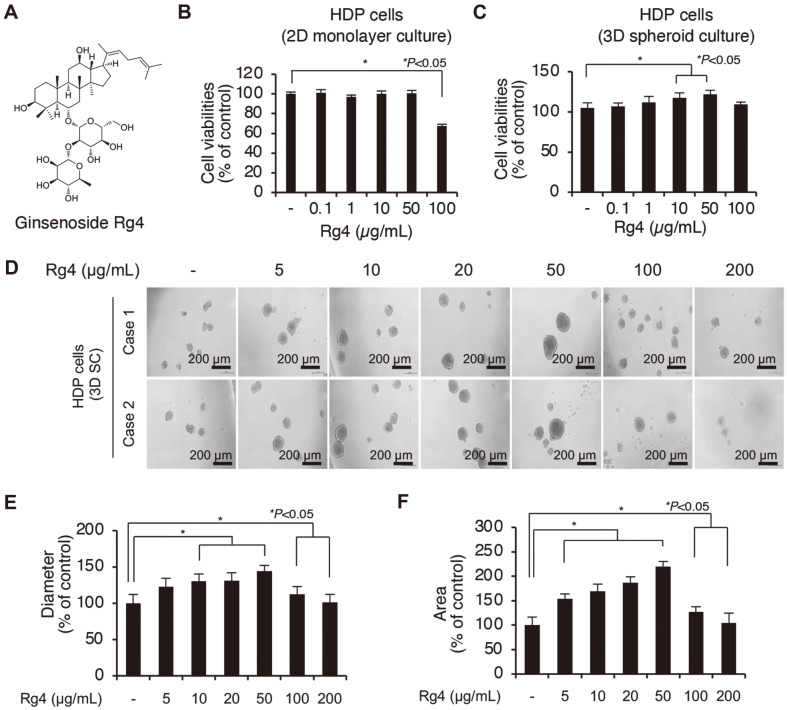
Rg4 increases cell viability in 3D-cultured HDP cells. (**A**) Chemical structure of ginsenoside Rg4. (**B**, **C**) Twoand three-dimensional cultured dermal papilla cells were treated with Rg4 (5-100 μg/ml) for 48 hours. Viability of DP cells were carried out by WST-1 assay. (**D**) Images showed DP sphere size which treated with Rg4 (5-200 μg/ml) for 48 hours. (**E**, **F**) The diameter and area of the DP spheres size was showed by graph through captured image. The results were presented as the mean ± SD of three independent experiments. *p < 0.05 versus DMSO-treated control. Scale bars: C, 200 μm.

The molecular structure of Rg4 is depicted in Fig. 1A. It has been reported that the ability of DP cells to form spheres is directly related to their increased viability [29]. Therefore, we first investigated whether Rg4 promotes the viability of DP cells. In order to compare the effect of Rg4 on the viability of DP cells, we prepared 2D monolayer cultures and 3D spheroid cultures of DP cells. For preparing the cultured DP spheres, the cells were maintained on a low-attachment, round-bottomed microplate and incubated for allowing self-assembly and sphere formation. The DP cells in the 2D and 3D cultures were treated with Rg4 at the indicated doses (0–100 μg/ml) for 24 h (Figs. 1B and 1C), and were subjected to WST-1-based cell viability analysis. As depicted in Fig. 1B, treatment with Rg4 did not affect cell viability up to a dose of 50 μg/ml. However, treatment with higher doses of Rg4 significantly decreased the viability of the DP cells in 2D culture. In contrast, we found that treatment of the DP cells in 3D culture with Rg4 significantly increased cell viability up to a dose of 50 μg/ml in a dose-dependent manner, compared to that of the DMSO-treated control. Based on the observations that the Rg4 ginsenoside increased the viability of the DP spheres (Fig. 1C), we next investigated the effect of Rg4 on inducing hair growth by comparing the size of the DP spheres. Previous studies have demonstrated that DP spheroids possess the hair-inductive properties of intact dermal papillae, and the sizes of the spheres correlate with the hair-inductive properties of DP cells [2]. To this end, the cells were treated with Rg4 at the indicated doses (0–200 μg/ml) using a 3D spheroid culture system. The cells were subsequently subjected to microscopic examination and the diameter of the spheres was analyzed. Treatment with Rg4 (0–50 μg/ml) gradually increased the size of the DP spheres compared to that of the DMSO-treated controls (Figs. 1D-1F and Supplementary Figs.). These results suggested that Rg4 promoted the proliferation and the formation of DP spheres.

### Ginsenoside Rg4 Increases Expression of Signature Genes in DP Spheres

**Fig. 2:**
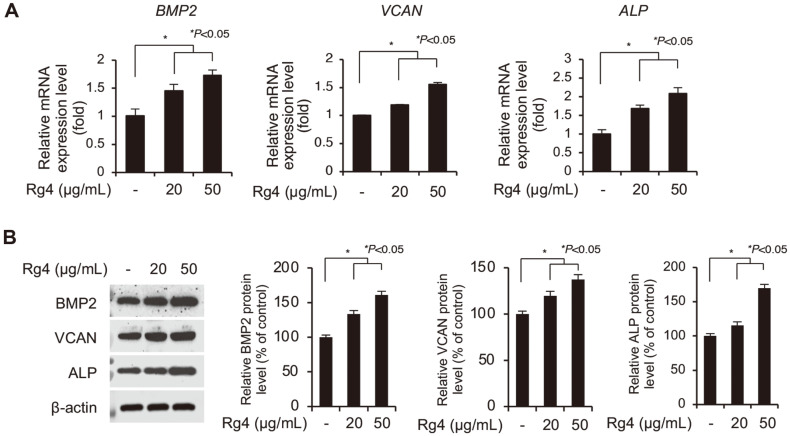
Rg4 increases hair growth-related signature gene expression in 3D spheroid cultured DP cells. (**A**) Dermal papilla cells were treated with Rg4 (20 and 50 μg/ml) for 48 h. The mRNA expression of DP signature genes, BMP2, VCAN and ALP, were analyzed by RT-qPCR and normalized against GAPDH. (**B**) The protein levels of *BMP2*, *VCAN* and *ALP* were changed by Rg4 treatment. β-actin was used as a loading control. The immunoblotting results were analyzed using the Image-J program. The data were presented as the mean ± SD of three independent experiments. Values of *p < 0.05 were considered to be statistically significant.

As depicted in Fig. 1, Rg4 increased the viability and size of the DP spheroids in 3D culture. In order to verify the effects of Rg4 on the hair-inductive properties of the DP spheres, we next investigated the effect of Rg4 on the expression levels of signature genes in DP cells, including *BMP2*, *VCAN*, and *ALP*. This was based on the observation that the formation of the spheres was accompanied by the restored expression of DP signature genes that produce important regulators which stimulate the hair-inductive properties of DP [2]. The expression of the DP signature genes in DP spheres treated with 20 or 50 μg/ml was analyzed using RT-qPCR. As depicted in Fig. 2A, treatment with Rg4 significantly upregulated the mRNA transcripts of the DP signature genes in a dose-dependent manner. In order to confirm these results, the protein expression levels of the DP signature genes in spheres treated with (20 or 50 μg/ml) Rg4 were measured by immunoblotting with the specific antibodies. Treatment with Rg4 also significantly increased the levels of these proteins in the DP spheres (Fig. 2B). In addition, the effect of Rg4 on cell proliferation was further confirmed by analyzing the expression levels of cell proliferation-related proteins, BCL2 and BAX in DP cells. Also, the effects of Rg4 on the expression on ALP, BMP2 and VCAN on DP cells were additionally confirmed using minoxidil as a positive control. The results showed that the increased levels of BCL2, and decreased levels of BAX were induced by Rg4 treatment (Supplementary Fig.). These results suggested that treatment with Rg4 induced the expression of DP signature genes in the DP spheres.

### Ginsenoside Rg4 Activates AKT/GSK3β Signaling Pathway in DP Spheres

**Fig. 3:**
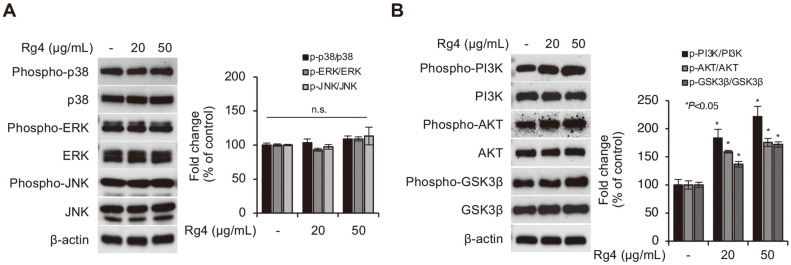
Treatment of Rg4 enhances the activation of PI3K/AKT/GSK3β signaling in 3D spheroid cultured HDP cells. DP cells were treated with Rg4 (20 and 50 μg/ml) for 48 h. The protein expression related (**A**) MAPK and (**B**) PI3K/ AKT/GSK3β signaling pathway was determined through immunoblotting assay. β-actin was used as a loading control. The bands were analyzed using the Image-J program and the date were presented as the mean ± SD of three independent experiments. Values of *p < 0.05 were considered to be statistically significant. N.S. means none significant.

The PI3K/AKT and mitogen-activated protein kinase (MAPK) signaling pathways are well-known mediators of cell signaling, leading to the activation of various cellular processes, including cellular survival and death in several cell types [30, 31]. In particular, the PI3K/AKT and MAPK (p38, ERK, and JNK) signaling cascades are reported to be important pathways involved in the proliferation and hair-inductive properties of DP cells [32]. We therefore hypothesized that the promotive effects of Rg4 on the enhanced hair-inductive properties of DP cells are mediated via the PI3K/AKT and MAPK signaling pathways in DP spheres. The expression levels of the proteins involved in the AKT and MAPK pathways were measured by immunoblotting. Any alterations in the molecules associated with the MAPK pathway in the DP spheres treated with Rg4 were consequently identified (Fig. 3A). The results demonstrated that treatment with Rg4 increased the phosphorylation of PI3K and AKT proteins in the DP spheres (Fig. 3B). We additionally observed that treatment with Rg4 increased the phosphorylation of GSK3β, an immediate target of AKT kinase, indicating that the activity of phospho-GSK3β was inhibited by the phosphorylation of serine 9 in the Rg4-treated DP spheres. These results suggested that the effect of Rg4 on the formation of DP spheres could be mediated via the activation of the AKT/GSK3β signaling pathway.

### Ginsenoside Rg4 Enhances WNT/β-Catenin Signaling Pathway in DP Spheres

**Fig. 4:**
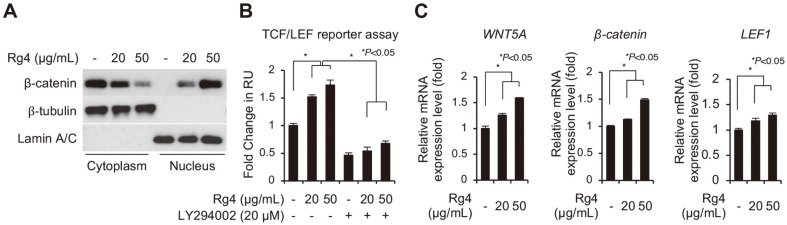
Rg4 upregulates hair-inducing activity via WNT/β-catenin signaling pathway in DP spheres. (**A**) β- catenin translocation was analyzed by immunoblotting assay in Rg4 treatment DP spheres. Lamin A/C and β-tubulin were used as loading control for total protein. (**B**) The luciferase activity of TCF/LEF reporter plasmid was confirmed by luciferase assay. DP cells were treated with Rg4 (20 and 50 μg/ml) with or without LY294002 (20 μM) for 48 hours. The luciferase assay was determined by normalizing the β-galactosidase activity. (**C**) The mRNA expression level of target genes including *WNT5A*, *β-catenin*, and *LEF1* were confirmed by RT-qPCR analysis. DP cells were treated with the indicated doses of Rg4 (20 and 50 μg/ml) for 48 h. The results are presented as the mean ± SD of three independent experiments. Values of *p < 0.05 were considered to be statistically significant.

In our previous study, we demonstrated that the phosphorylation of GSK3β at serine 9 by activated AKT plays a role in the hair-inductive properties of DP cells via the activation of Wnt/β-catenin signaling, which is one of the most important signaling pathways for hair growth in HFs [12]. We therefore investigated whether the activation of AKT kinase by Rg4 leads to the activation of the Wnt/β-catenin signaling pathway in DP spheres using immunoblotting analysis. The data obtained by immunoblotting revealed that the increased nuclear translocation of β-catenin in the DP spheres was enhanced by treatment with Rg4 in a dose-dependent manner (Fig. 4A). In order to further confirm the immunoblot data, we analyzed the transcriptional activity of β-catenin using a luciferase reporter assay. The DP cells were transfected with luciferase reporter plasmids containing TCF/LEF responsive elements controlled by β-catenin. The transfected DP cells were subsequently cultured in a 3D culture system, followed by treatment with Rg4. As depicted in Fig. 4B, treatment with Rg4 increased the luciferase activity in the DP spheres in a dose-dependent manner. We additionally observed that the increase in the luciferase activity was abolished by treatment with LY294002, a potent PI3K/AKT inhibitor, indicating that the activation of β-catenin signaling in DP cells by Rg4 was dependent on P13K/AKT signaling. Finally, we confirmed the changes in the mRNA expression of the target downstream genes of WNT/β-catenin, including *WNT5A*, *β-catenin*, and *LEF1* in DP spheres treated with Rg4, by RT-qPCR analysis. The results revealed that treatment with Rg4 significantly increased the mRNA expression of the target genes in DP spheres compared to that of the controls, indicating that Rg4 activated Wnt/β-catenin signaling in the DP spheres (Fig. 4C). Taken together, our results suggested that Rg4 promotes the hair-inductive properties of the DP spheres via the AKT/GSK3β/β-catenin signaling cascade.

## Discussion

Hair loss or alopecia is one of the foremost dermatological disorders worldwide, and is caused by multiple factors, including immunological abnormalities, genetics, hormones, environmental factors, medications, and nutrition [33]. Alopecia affects both men and women and can occur as a result of alterations in the modified or shortened hair cycle, HFs, or the shafts [6]. Alopecia is also considered to be a psychosomatic disease because patients with alopecia may suffer from several psychosocial problems, including low self-esteem, high levels of depression, and anxiety [34]. Increasing evidence on the pathogenesis of alopecia has demonstrated that 5α-dihydrotestosterone (5α-DHT) binds to the androgen receptor on DP cells resulting in progressive follicular miniaturization, shortening of the anagen phase, and hair loss [6, 7]. Additionally, numerous signaling pathways, including the stimulatory pathways, such as the Wnt/β-catenin, STAT3, and Shh pathways, as well as the inhibitory pathways including Dkk-1, BMP4, and Dickkorpf-related protein are implicated in the treatment of alopecia [35]. However, there are only two medications for hair loss at present, namely, minoxidil and finasteride [35]. Minoxidil is indicated for the treatment of androgenetic alopecia, which is the most common type of alopecia in men and women. A previous study demonstrated that minoxidil prolongs the anagen phase in the DP by inducing Wnt signaling and stimulating follicular proliferation and differentiation [36]. Finasteride has been approved as an anti-androgenic agent for men with male-pattern hair loss [37]. This medication inhibits the expression of 5α-reductase, which regulates the production of 5α-DHT from testosterone [37]. However, these treatments for alopecia have limited therapeutic efficacy [38], which makes it necessary to understand the exact mechanism underlying hair growth and hair loss, and develop alternative agents for the treatment of hair loss without any side effects. The results of our study suggested that ginsenoside Rg4 can serve as an alternative natural therapy for hair loss.

In this study, we report for the first time that ginsenoside Rg4 plays a potential role in promoting hair growth by enhancing the hair-inductive property of DP spheroids without inducing cytotoxicity. Ginsenosides are natural components of ginseng and have been suggested as promising candidates for the treatment of hair loss. Numerous studies have reported that the extracts and ginsenosides of ginseng promote hair growth by inducing the stimulation of outer root sheath (ORS) and human dermal papilla cells (hDPC) and increasing the growth phase of the hair cycle [24, 39]. Studies in murine models have also demonstrated that the ginsenoside F2 promotes the anagen phase of hair growth by activating the Wnt signaling pathway [40]. Furthermore, existing reports demonstrate that the extracts of ginseng and its ginsenosides including Rb1, Re, and Rg1 promote hair growth and their effects are similar to that of minoxidil [41]. It has been additionally demonstrated that several ginsenosides affect hair growth by enhancing the hair-inductive abilities of DP cells [39, 42, 43]. However, one limitation of ginsenosides, including ginsenosides Rb1 and Rg1, is that their bioavailability is very low [44]. Previous studies have demonstrated that the bioavailability of these ginsenosides is increased by their transformation to low molecular weight metabolites (rare ginsenosides) including their deglycosylated forms [45]. This indicated that Rg4 is a rare ginsenoside, and the results of our study present a novel strategy for promoting the hair-inductive property of DP cells.

In this study, we observed that Rg4 promotes the inductive properties of DP spheres on hair growth when cultured in a 3D-spheroid culture system. DP cells are an attractive experimental system for studying the inductive signals necessary for hair growth. This is due to the fact that DP cells are the main mesenchymal components necessary for hair growth, and play a pivotal role in inducing the formation of new HFs [5]. However, DP models of 2D monolayer culture are correlated with the loss of expression of specific genes and reduced hair-inductive activity [15]. Recent studies have focused on developing an alternative in vitro DP model for rescuing the hair-inductive ability of DP cells [46]. Indeed, DP spheres cultured under 3D culture conditions are morphologically similar to intact papilla, and rescue the hair-inductive properties of DP cells [15, 46]. Another study reported that the potential inductive effects on hair growth is partially recovered in the model of DP spheroids in 3D culture and demonstrated that these spheroids can induce the growth of new hairs [2]. The present study demonstrated an increase in both the viability and size of the DP spheres in the 3D culture system following treatment with Rg4. The diameter and area of DP spheroids were significantly increased with various doses, specifically, 5, 10, 20, and 50 μg/ml of Rg4. These results indicated the dose-dependent effect of Rg4 on cell proliferation and spheroid formation, not a nonspecific effect of Rg4 on these results. Furthermore, 100 μg/ml of Rg4 showed some toxicity on DP cells; however, the toxic effect was observable only in 2D (monolayered)-cultured DP cells, not a 3D DP spheroid. In addition, the DP cells used in this study are isolated from a single human tissue, and the excessive interpretation of the toxicity and effects of Rg4 is rather unreasonable. Therefore, further in-depth studies of the toxicity and clinical evaluation are needed to better understand the effect of Rg4 treatment in hair growth induction. The study additionally demonstrated that Rg4 regulated the expression of DP signature genes including *BMP*, *VCAN*, and *ALP*. These genes are upregulated in DP cells during the anagen phase of hair growth, and are considered as active indicators of the inductive ability of DP cells on hair growth [3, 47, 48]. The results of this study indicated that Rg4 can enhance the hair-inductive effect of DP cells by regulating the properties of DP cells, including their aggregative behavior and expression of DP signature genes.

Investigation of the mechanism underlying the regulatory effect of Rg4 on the hair-inductive properties of DP cells revealed that treatment with Rg4 enhanced the activation of AKT and increased the phosphorylation of its downstream target, GSK-3β. The activation of PI3K/AKT signaling following treatment with Rg4 has been investigated in a previous study [27]. Studies analyzing the effect of ginseng extracts on hair growth have demonstrated that AKT signaling is essential for the proliferation of DP cells [24]. It has been additionally demonstrated that the activation of AKT plays an important role in the inductive effects of DP cells on hair growth [12, 49]. The results of this study are consistent with those of previous studies in that it was observed that treatment with Rg4 induced the activation of AKT and the expression of GSK-3β, indicating that Rg4 regulates the hair-inductive property of DP spheres via the AKT/GSK-3β signaling pathway. Furthermore, the inhibitory phosphorylation of GSK-3β in the DP spheres was stimulated by treatment with Rg4, suggesting that the downstream targets of GSK-3β were activated by treatment with Rg4. Previous studies have demonstrated that the inhibition of GSK-3β rescues from degradation and leads to the activation of the WNT signaling pathway, which plays a critical role in the morphogenesis of HFs and the induction of the anagen phase [11, 12, 24]. As expected, the activity of β-catenin, a key downstream effector of GSK-3β, increased in the Rg4-treated DP spheres, and the activity of β-catenin was abolished by treatment with the AKT inhibitor, LY294002. These results indicated that the enhanced hair-inductive ability of DP cells following treatment with Rg4 is mediated via the AKT/GSK-3β/β-catenin signaling pathway. In order to ensure that the WNT signaling pathway is activated by treatment with Rg4, we further confirmed that the expression of the target genes of the WNT signaling pathway including *WNT5A*, *β-catenin*, and *LEF1* was enhanced by treatment with Rg4. These results indicated that Rg4 could serve as an effective natural therapy for hair loss by activating the hair-inductive ability of DP cells via the activation of the AKT/GSK-3β/β-catenin signaling pathway.

The hair follicle is composed of dermal papilla cells, specialized mesenchymal cells, and keratinocytes, the epithelial cells that comprise the hair shaft, the encircling inner and outer root sheaths [5]. Therefore, in hair growth, not only the proliferation of DP cells but also the influence on keratinocyte are important [4]. During the anagen phase, proliferation of DP cells is activated, which increases the DP size of the hair bulb [5]. These effects are enhanced by activating Wnt/β-catenin signaling pathway in DP cells [50] and the growth of hair follicle is grown by the influence of various growth factors, including VEGF, IGF-1 and KGF, secreted from DP cells [4, 5, 50]. These growth factors also affect the proliferation and differentiation of epithelial components, especially keratinocytes, to generate new follicle and growth of hair shaft [5]. Here, we found that Rg4 increased the proliferation and spheroid size of DP cells and these effects were mediated by AKT/GSK3β signaling pathway. In addition, we found that these effects were followed by nucleus translocation of β-catenin and the expression of its downstream targets including WNT5A. Meanwhile, VEGF and IGF-1 are the transcription target genes of β-catenin/TCF-LEF complex [51, 52]. Our previous study showed that activation of AKT/GSK3β/ β-catenin signaling pathways in DP cells increased the expression level of VEGF and IGF-1 and those results enhanced the proliferation and chemotactic migration of HaCaT keratinocytes [12]. These results indicate that activation of Wnt/β-catenin signaling pathway in DP cells is at least in part related to expression and secretion of VEGF and IGF-1 factors that are important regulator of proliferation and differentiation of keratinocytes on hair growth. Taken together, the activation effect of Rg4 on Wnt/β-catenin signaling pathway in DP cells may affect the proliferation and differentiation of keratinocyte on hair bulk.

In summary, treatment with Rg4 promoted the inductive effect of DP cells on hair growth by enhancing sphere formation and upregulating the expression of DP signature genes via the activation of the AKT/GSK3β/β-catenin signaling pathway. Moreover, Rg4 could serve as an alternative natural therapy for hair loss. Our findings suggest that ginsenoside Rg4 can be used as a potential candidate for the treatment of hair loss.

## Supplemental Materials



Supplementary data for this paper are available on-line only at http://jmb.or.kr.

## Figures and Tables

**Table 1:** List of sequences used for RT-qPCR.

Targets of RT-qPCR	Sequence of primer
Bone morphogenetic protein 2 (*BMP2*)	F : 5’- GGAACGGACATTCGGTCCTT -3’
	R : 5’- CACCATGGTCGACCTTTAGGA -3’
Versican (*VCAN*)	F : 5’- TGTCCGATTCATAGTCCTGTC -3’
	R : 5’- CTCACAGCGATAAGTGCCCTC -3’
Alkaline phosphatase (*ALP*)	F : 5’- CAAACCGAGATACAAGCACTC -3’
	R : 5’- CGAAGAGACCCAATAGGTAGTCCAC -3’
WNT family member 5A (*WNT5A*)	F : 5’- TTGAAGCCAATTCTTGGTGGTCGC -3’
	R : 5’- TGGTCCTGATACAAGTGGCACAGT -3’
Lymphoid enhancer binding factor 1 (*LEF1*)	F : 5’- GCTGTCTTTCTTTCCGTGCTA -3’
	R: 5’- GCTGTCTTTCTTTCCGTGCTA -3’
β-catenin	F : 5’- CCCACTAATGTCCAGCGTTT -3’
	R : 5’- AACCAAGCATTTTCACCAGG -3’
Glyceraldehyde-3-phosphate dehydrogenase (*GAPDH*)	F : 5’- CGGAGTCAACGGATTTGGTCGTAT -3’
	R : 5’- AGCCTTCTCCATGGTGGTGAAGAC -3’
