# Supplementary material for: Ginsenoside Rg4 Enhances the Inductive Effects of Human Dermal Papilla Spheres on Hair Growth Via the AKT/GSK-3β/β-Catenin Signaling Pathway
Source: J Microbiol Biotechnol. 2021 May 31;31(7):933–41. doi: 10.4014/jmb.2101.01032 (PMC9706015; doi:10.4014/jmb.2101.01032)
Supplement: Supplementary file 1 [file jmb-31-7-933-supple.pdf]

## Supplementary Figure

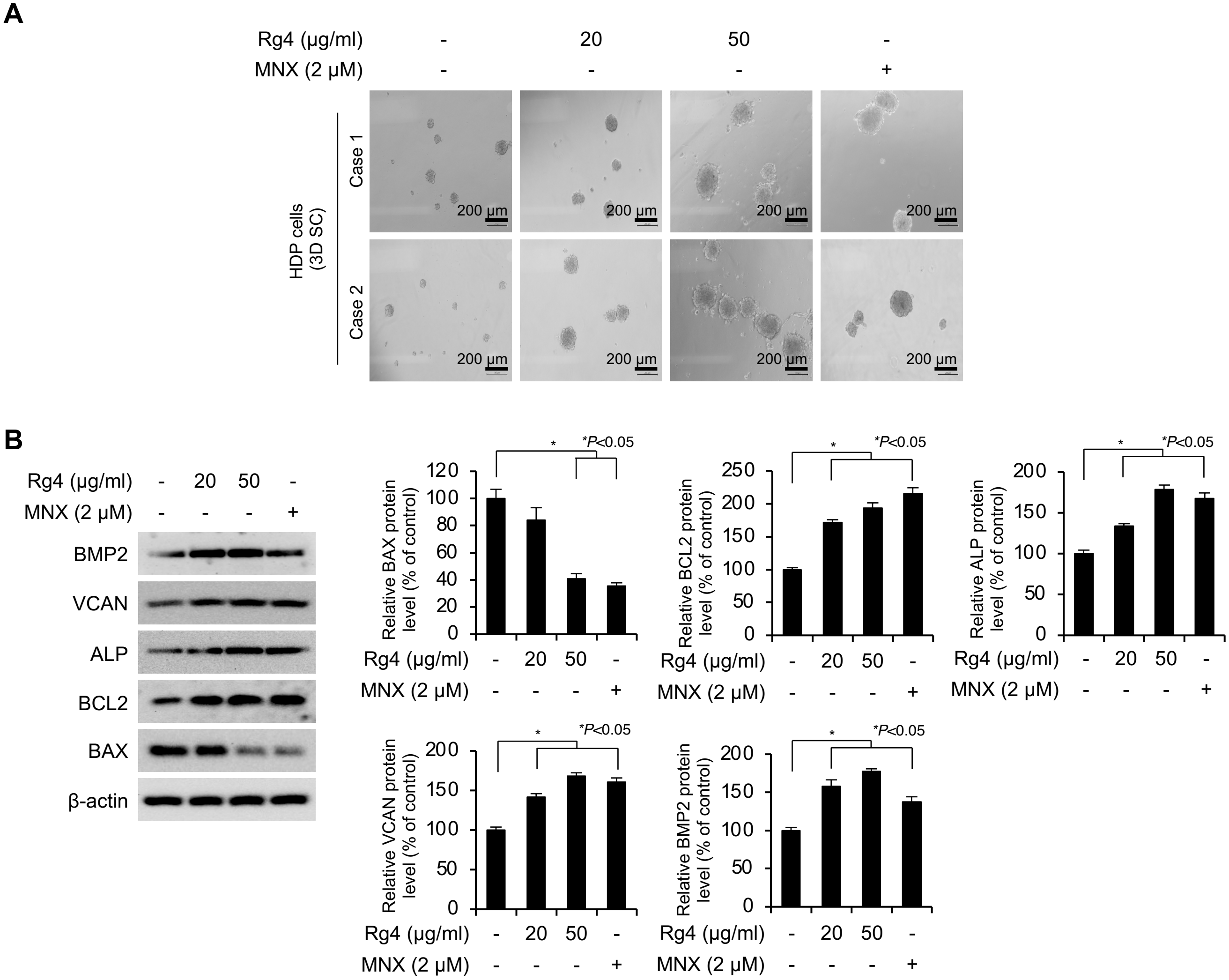


**Supplementary Figure.** Rg4 regulates cell growth and hair growth-related signature protein expression in 3D spheroid cultured DP cells.

(A) Images showed DP sphere size which treated with Rg4 (20 and 50 μg/mL) for 48 hours. (B) Dermal papilla cells were treated with Rg4 (20 and 50 μg/mL) for 48 hours. The protein levels of BMP2, VCAN, ALP, BCL2 and BAX were changed by Rg4 treatment. β-actin was used as a loading control. Minoxidil (MNX) was used as a positive control. The immunoblotting results were analyzed using the Image-J program. The data were presented as the mean ± SD of three independent experiments. Values of $*P < 0.05$ were considered to be statistically significant.
